# Complex dynamics in recurrent cortical networks based on spatially realistic connectivities

**DOI:** 10.3389/fncom.2012.00041

**Published:** 2012-07-10

**Authors:** N. Voges, L. Perrinet

**Affiliations:** Institut des Neurosciences de la Timone (INT), Aix-Marseille Université, CNRS (UMR 7289)Marseille, France

**Keywords:** cortical network dynamics, local and random remote connections, phase space differences

## Abstract

Most studies on the dynamics of recurrent cortical networks are either based on purely random wiring or neighborhood couplings. Neuronal cortical connectivity, however, shows a complex spatial pattern composed of local and remote patchy connections. We ask to what extent such geometric traits influence the “idle” dynamics of two-dimensional (2d) cortical network models composed of conductance-based integrate-and-fire (iaf) neurons. In contrast to the typical 1 mm^2^ used in most studies, we employ an enlarged spatial set-up of 25 mm^2^ to provide for long-range connections. Our models range from purely random to distance-dependent connectivities including patchy projections, i.e., spatially clustered synapses. Analyzing the characteristic measures for synchronicity and regularity in neuronal spiking, we explore and compare the phase spaces and activity patterns of our simulation results. Depending on the input parameters, different dynamical states appear, similar to the known synchronous regular “SR” or asynchronous irregular “AI” firing in random networks. Our structured networks, however, exhibit shifted and sharper transitions, as well as more complex activity patterns. Distance-dependent connectivity structures induce a spatio-temporal spread of activity, e.g., propagating waves, that random networks cannot account for. Spatially and temporally restricted activity injections reveal that a high amount of local coupling induces rather unstable AI dynamics. We find that the amount of local versus long-range connections is an important parameter, whereas the structurally advantageous wiring cost optimization of patchy networks has little bearing on the phase space.

## 1. Introduction

Typically, the study of cortical network dynamics was based on randomly connected single-cells without any spatial embedding (e.g., Amit and Brunel, 1997; van Vreeswijk and Sompolinsky, 1998; Brunel, 2000; Kumar et al., 2008a). Likewise, neural mass (or neural field) models mostly assumed homogeneous random intrinsic couplings whereas the external links between different masses were more specifically chosen (Jansen and Rit, 1995; Deco et al., 2011). Alternately, several studies on single-cell networks turn to local distance-dependent connectivity assumptions describing the dynamics within the range of a so-called cortical column of about one millimeter radius (Mehring et al., 2003; Kumar et al., 2008b; Kriener et al., 2009; Yger et al., 2011). It turns out that there are important functional advantages of including such local connectivity, e.g., for the retrieval of states in associative memory (Roudi and Treves, 2004) or with respect to orientation selectivity (Buzás et al., 2006).

In reality, however, intrinsic cortical synapses are established with respect to various features, as, for instance, cell-type specificity (e.g., preferred couplings between certain neurons and/or layers, see White, 2002), and the spatial distance between the neurons. Even inside the gray matter, i.e., with respect to axons that do not enter the white matter, there is a distinction between local and remote connections (Kisvárday and Eysel, 1992; Voges et al., 2010a,b). The local connection probability is usually approximated by a Gaussian distance-dependent decay (Buzás et al., 2006; Hellwig, 2000; Stepanyants et al., 2008), while non-local projections are often confined to a limited number of discrete spots in space, the so-called “patches” (e.g., Kisvárday and Eysel, 1992; Binzegger et al., 2007; Voges et al., 2010a,b). Thus, cortical connectivity is neither merely random nor confined to local couplings; in fact, it is a combination of both (Braitenberg and Schüz, 1998; Stepanyants et al., 2009; Voges et al., 2010a,b).

Accordingly, several large-scale neural mass (or neural field) models include spatially modulated connectivity kernels (e.g., Troy and Schusterman, 2007; Kilpatrick and Bressloff, 2010). In particular from the wiring cost perspective, such a combination of local and remote couplings is highly advantageous (Buzsaki et al., 2004; Chklovskii, 2004; Voges et al., 2010b). On one hand, signal propagation needs to be fast and efficient, even between distant neurons. On the other hand, the longer axon collaterals are, the more space they take up, and space is limited inside the skull. Therefore, small-world networks are an attractive approach for cortical network models (Newman, 2003; Voges et al., 2012b): they span the whole range between (local) regular and random connectivity by varying one single parameter which characterizes the number of regular local versus long-range connections.

In our study we ask for the consequences on the dynamics if discrete cortical networks comprise such real-world connectivity patterns, i.e., a mixture of local and remote couplings, as well as patchy projections. To this end, we require an extended spatially embedded two-dimensional (2d) network that enables us to consider such connectivity patterns going beyond the range of a cortical column. Our network models consist of two distinct types of conductance-based integrate-and-fire (iaf) neurons (Nowak et al., 2003) whose connection probabilities are adapted to the cat's visual cortex (Binzegger et al., 2004). Given these assumptions, we analyze the effect of different network structures on their “idle” dynamics. Having started with purely random connectivity in (Voges and Perrinet, 2010), we here advance to various types of mixed connectivity structures, including patchy projections. In addition to the one extreme of merely random connections, we also consider the other extreme, i.e., a network where all synapses are established as local distance-dependent projections. As all these architectures are based on identical fundamental parameters (**Sections 2.1** and **3**), we are in a position to analyze the isolated effect of their structural differences. We investigate if certain connectivity assumptions alter the appearance, the position, and/or the sharpness of phase space transitions, as well as possible changes in terms of occurring activity patterns. Moreover, to take into account the stability of the resulting dynamical states, we also investigate the effect of additional localized activity injections.

Initially, Brunel (2000) demonstrated the existence of different dynamical states for sparsely connected random networks composed of 80% excitatory (exc.) and 20% inhibitory (inh.) single neurons. Depending on the ratio between the strength of exc. and inh. synaptic weights and the external input rate, the ensemble of all neurons fired either synchronously or asynchronously, and regularly or irregularly. Kumar et al. (2008a) considered conductance-based synapses instead of current-based synapses, leading to significant differences in their phase space compared to (Brunel, 2000). Roxin et al. (2005) showed that including conduction delays clearly affects the resulting dynamics of 1d ring graphs. Assuming 2d spatially embedded random networks (Voges and Perrinet, 2010), we demonstrated the emergence of a new critical parameter, as well as yet another set of phase space changes which were at least partially based on the inclusion of distance-dependent conduction delays.

Several other studies investigate the relationship between structural network properties and their dynamical consequences. For instance, Kitano and Fukai (2007) studied the variability and synchronicity in neuronal spiking in dependence of the rewiring probability in 2d networks based on a small-world topology. Recently, Yger et al. (2011) analyzed the dynamics of balanced 2d locally connected networks of conductance-based iaf neurons for varying Gaussian connectivity profiles. They found that the macroscopic properties of the spiking activity are invariant with respect to their different connectivity assumptions. Kriener et al. (2009), however, showed that strong common input, e.g., induced by predominant neighborhood coupling, amplifies synchrony in recurrent networks. In contrast, a broad degree distribution enhances fluctuations in the spike rates, and it shapes the power spectrum of the population activity by partially destroying the global oscillations in certain frequency bands (Denker et al., 2004; Tetzlaff et al., 2008). Similarly, Roxin (2011) investigated the effect of broadening binomial degree distributions on the network dynamics. Compared to such studies on specific details, we focus on actually existent cortical projections patterns as a whole, rather similar to Kitano and Fukai (2007) and Yger et al. (2011) but on a larger spatial scale. It is the aim of this paper to provide first insights into the dynamical consequences of different connectivity structures for a spatially extended network model, using the neuroanatomically realistic parameters detailed in Voges et al. (2010a,b).

In the following **Section 2**, we explain the different network structures and connectivity profiles considered in this study. Important parameters and measures used to simulate and characterize the network dynamics are summarized in **Section 3**. Then, we present the results of our simulations in **Section 4**. We finish off with a discussion of our results.

## 2. Network connectivity structures

This section describes the five different network architectures whose dynamics are to be analyzed in this study and some of their neuroanatomical background. In order to allow for non-local synapses, we assume a 2d cortical sheet of 5 × 5 mm^2^, a relatively large spatial region compared to previous studies, which typically represent one squared millimeter (Mehring et al., 2003; Kumar et al., 2008a). Thus, we focus on connectivity patterns that occur on a spatial scale that comprehends many cortical columns. According to Buzsaki et al. (2004); Binzegger et al. (2007) both exc. and inh. neurons establish non-local synapses. Remote inh. projections, however, are less frequent with a much shorter spatial range than remotely established synapses of exc. neurons (Binzegger et al., 2007). We distinguish between the following network architectures:

- Randomly connected networks (RD): In this basic model the synapses of each neuron are generated randomly, i.e., independently of the spatial positions of the cells. The resulting network and its phase space dynamics have already been presented in (Voges and Perrinet, 2010). Nevertheless, we include this model as default network to estimate the effect of altered connectivities.
- Locally connected networks (LO): Here we assume that all synapses of all neurons are established locally within the neighborhood of each neuron. This local connectivity profile is characterized by a Gaussian distance-dependent decay (Stepanyants et al., 2008; Hellwig, 2000), see **Section 2.1**. Note, however, that other studies find that this distance dependence is better described by an exponential decay (Holmgren et al., 2003) and that distribution of synaptic efficaciesis is skewed and not Gaussian (Song et al., 2005).
- Mixed connectivities: Each neuron targets both neurons situated within its local neighborhood and remotely located neurons (Kisvárday and Eysel, 1992; Binzegger et al., 2007; Voges et al., 2010a,b). The local connectivity profiles of these three network models are identical, however, their connectivities differ with respect to the spatial arrangement of the remote projections.
  – Homogeneously distributed remote synapses (RM): In addition to its local couplings each neuron projects to randomly chosen remote cells, resulting in a spatially homogeneous distribution of the distant targets. On one hand, this type of mixed connectivity represents the simplest modeler assumption. On the other hand, it agrees with the findings of (van Hooser et al., 2006) who claim the absence of patchy connections in the primary visual cortex of mammals without orientation maps.
  – Patchy remote connections (OP, PB): In addition to its local couplings, each neuron projects to spatially clustered remote cells as typically suggested for cortical long-range projections (Kisvárday and Eysel, 1992; Lewis et al., 2002; Angelucci and Bressloff, 2006; Buzás et al., 2006; Binzegger et al., 2007; Voges et al., 2010a,b). We consider two different patchy projection patterns: OP and PB. The rather deterministic OP model assumes a partial overlap of the termination fields of neighboring neurons. The resulting projection pattern of a cigar-shaped group of adjacent neurons is similar, e.g., to the elongated stripes resulting from extracellular tracer injections in the monkey prefrontal cortex (Lewis et al., 2002; Voges et al., 2010a,b). In the PB model we consider the set of all synaptic targets of groups of neurons that are situated in spatially confined regions (boxes). All neurons in such a box establish a common patchy projection pattern, whereas each single neuron projects into a randomly chosen subset of these patches. As explained in (Voges et al., 2010a,b) this is the most appropriate projection pattern if one aims to reproduce neuroanatomical findings with respect to both intra- and extra-cellular tracer injections.

**Figure 1:**
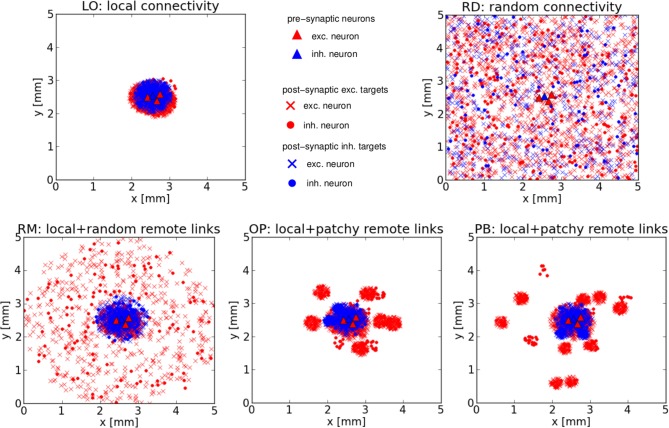
**Network structures.** Typical connectivity for the five network architectures from an exemplary realization of our 5 × 5 mm^2^ sheet of cortex. Each image shows the positions of all pre- and post-synaptic cells of three excitatory and one inhibitory neuron, located near the center of each square. **Top row:** Purely local (LO) or random (RD) connections. **Bottom row:** The three mixed architectures comprising local and random remote (RM) or local and patchy remote (OP, PB) connections.

Figure 1 shows the typical connectivity of the five network architectures for three exc. and one inh. cell chosen randomly (from the central region) of an exemplary realization of the 5 × 5 mm^2^ sheet of cortex. Presynaptic exc. (inh.) cells are represented by red (blue) triangles, exc. (inh.) postsynaptic targets are represented by red (blue) crosses if the presynaptic neuron is an exc. cell, or red (blue) disks if the presynaptic neuron is an inh. cell.

### 2.1. Details on network connectivities

The general settings and parameters briefly repeated in this section are identical to those used in (Voges and Perrinet, 2010). For all network structures, we consider *N* = 49, 163 neurons^1^ that are spatially embedded in a 2d quadratic domain of side length *R* = 5 mm. The assumption of periodic boundary conditions leads to a maximum projection range of *r*_max_ = 2.5 mm. Following Binzegger et al. (2004) and focusing on layer 2/3 of the cat's visual cortex, we assume 22% inh. cells, i.e., *N*_*i*_ = 104^2^ neurons arranged on jittered lattice positions. For the remaining 78% exc. pyramidal or spiny stellate cells, we assume uniformly distributed spatial positions. The global connectivity of all network models analyzed in this study is $c=\bar{k}/N\approx 0.0153$, with an average number $\bar{k}≃752$ in- and out-going synapses per neuron, respectively. Note that usually, the numeric relation between the number of exc.–exc., exc.–inh., inh.–exc., and inh.–inh. synapses exclusively depends on the frequencies of exc. and inh. neurons (Amit and Brunel, 1997; van Vreeswijk and Sompolinsky, 1998; Brunel, 2000; Kumar et al., 2008a). In contrast, and again following Binzegger et al. (2004), our networks comprise 71.1% exc. to exc. (β_*ee*_ = 0.711), 9.96% exc. to inh. (β_*ei*_ = 0.0996), 16.14% inh. to exc. (β_*ie*_ = 0.1614), and 2.8% inh. to inh. (β_*ii*_ = 0.028) synapses.

For four out of the five network models we analyze, we distinguish between local and remote connections. The global connectivity is thus composed of the following terms:

(1) $$c={\text{β}}_{ee}\cdot c+{\text{β}}_{ei}\cdot c+{\text{β}}_{ie}\cdot c+{\text{β}}_{ii}\cdot c$$

(2) $$=:\left(c_{ee}^{\mathrm{loc}}+c_{ee}^{\text{rm}}\right)+\left(c_{ei}^{\text{loc}}+c_{ei}^{\text{rm}}\right)+\left(c_{ie}^{\text{loc}}+c_{ie}^{\text{rm}}\right)+\left(c_{ii}^{\text{loc}}+c_{ii}^{\text{rm}}\right)$$

Based on (Kisvárday and Eysel, 1992; Voges et al., 2010a,b), we assume that 60% of all out-going synapses of the exc. neurons in the network models with mixed connectivities (RM, OP, PB) are local ones. They are established according to a Gaussian distance-dependent profile (Hellwig, 2000; Stepanyants et al., 2008), see Equation (5). The maximum value of the connection probability between exc. neurons was set to *p*^max^_*ee*_ = 0.8 (Hellwig, 2000). The width of their Gaussian connectivity profile is defined as σ_*ee*_ = 0.25 mm. The latter value corresponds to the distance *d*_1/2_ = 0.24 mm for which *p*^max^_*ee*_ decays to ½ *p*^max^_*ee*_ as in Stepanyants et al. (2008). Thus,

(3) $$\begin{array}{cc} c_{ee}^{\text{loc}}=\frac{N_{e}^{2}}{N^{2}}\cdot 2\pi \frac{p_{ee}^{\max}\sigma_{ee}^{2}}{R^{2}}\cdot \langle 1-\exp\left(-\frac{bl^{2}}{2\sigma_{ee}^{2}}\right)\rangle & \text{with} \end{array}$$

(4) $$\begin{array}{c} \sigma_{ee}^{2}=\frac{d_{1/2}}{2\ln(2p_{ee}^{\max})} \end{array}$$

for a Gaussian connectivity profile according to

(5) $$p(d)=p^{\max}\cdot \exp\left(-\frac{d^{2}}{2\sigma^{2}}\right),$$

where *d* is the distance between any two neurons. Therewith, we fix the global connectivity to *c* = *c*_*ee*_/(β_*ee*_ · 0.6). The remaining connectivity parameters are determined with respect to this value, and are subject to the following additional assumptions:

- We define *p*^max^ ≤ 1 for all connections, i.e., we allow for only one synapse between any two neurons, and we do not permit autapses.
- According to Braitenberg and Schüz (1998), inh. neurons exhibit a slightly shortened local projection range with an increased number of synapses (due to stronger neuritic arborizations) as compared to exc. pyramidal cells. Hence, we assume σ_*ii*_ = 3/4 · σ_*ee*_ together with a corresponding increase in *p*^max^_*ii*_. The local connectivity range between exc. and inh. neurons is fixed to σ_*ei*_ = σ_*ie*_ = (σ_*ee*_ + σ_*ii*_)/2, together with the corresponding changes for *p*^max^_*ei*_ and *p*^max^_*ie*_.
- We assume 27% remotely established inh. synapses with a maximal spatial connectivity range of 0.7 mm, and a minimal distance of 0.25 mm.
- For the LO network model with only local couplings (c^rm^ = 0), the width of the Gaussian describing the connectivity between exc. neurons is increased to σ_*ee*_ = 0.33 mm. Keeping *c* constant, this results in *p*^max^_*ee*_ = 0.96. The other parameters are changed accordingly.

For the patchy network models (OP, PB) some additional parameters have to be defined. For a detailed derivation, based on neuroanatomical findings, see Binzegger et al., 2007; Voges et al., 2010a,b. The radius of the patches established by exc. and inh. neurons is set to be 0.2 mm and 0.15 mm, respectively. The radial distance from an inh. cell body to its patch is randomly chosen from [0.4, 0.55] mm, while the angle describing the spatial position of inh. patches is randomly chosen from (0, 360) degrees.

In the OP network model, each exc. (inh.) neuron establishes three (two) patches. The radial distance between an exc. cell body and each of its patches is drawn from a normal distribution with a mean of 1 ± 0.3 mm. Its angle is restricted to integer multiples of 60° (see Voges et al., 2010a).

In the PB model, the number of patches of exc. and inh. neurons is generated via binomial distributions: for inh. patches with a mean of two and a maximum of three, for exc. patches with a mean of three and a maximum of seven. In contrast to the OP model, the PB patch positions are determined for the set of all exc. neurons located in a box^2^. To each box, we assign 8–12 patches (uniform distribution), whose distances between the center of the box and the patch are drawn from one of two normal distributions, with means of 1 ± 0.2 mm and 1.7 ± 0.2 mm. These values are chosen according to (Buzás et al., 2006; Voges et al., 2010a,b).

The realizations of our five network architectures are based on identical spatial positions of the neurons. All networks with combined local and remote connections (mixed connectivities: OP, PB, RM) include identical local couplings.

## 3. Network dynamics

We now describe the dynamical properties and parameters assumed for simulating the dynamics of the discrete single-cell networks detailed above. We then list and explain the measures used to characterize the corresponding phase spaces and activity patterns.

We aim to investigate the effect of an increased structural complexity on the network dynamics while considering purely random connectivity as the default architecture to compare with. Therefore, the parameters characterizing single neuron dynamics, briefly summarized in the following paragraph, are identical to those described in (Voges and Perrinet, 2010). We consider conductance-based iaf neurons (Tuckwell, 1988; Gerstner and Kistler, 2003; Kumar et al., 2008a,b), adapted to represent two types of neurons: regular spiking exc. cells and fast spiking inh. cells (Brunel and Wang, 2003; Muller, 2003; Nowak et al., 2003). The spiking threshold *V*_θ_ = −55 mV, as well as the resting and reset potentials *V*_rest_ = *V*_rest_ = −70 mV, are identical for both neuron types. The synaptic time constants τ^*e*,*i*^ = 1.5 and 10 ms, the reversal potentials *V*^*e*,*i*^_rev_ = 0 and −80 ms, the membrane capacitances *C*^*e*,*i*^_*m*_ = 289.5 and 141 pF, and the membrane conductances at rest *G*^*e*,*i*^_rest_ = 29 and 21.2 nS are different for exc. and inh neurons, leading to distinct membrane time constants τ^*e*,*i*^_rest_ = *C*^*e*,*i*^_*m*_/*G*^*e*,*i*^_rest_ = 10 and 6.7 ms. We assume an average conduction velocity of 0.15 m/s for neurons located close to each other, and 0.3 m/s for distances larger than 1.5 mm (i.e., potentially myelinated axons) which results in a distance-dependent conduction delay for all internal synapses (Bringuier et al., 1999), in addition to a baseline value in the range [1.2, 1.5] ms. Excitatory neurons receive an external Poissonian input rate ν while the input to inh. neurons is reduced to ν × 0.66 (Voges and Perrinet, 2010).

Excitatory synaptic weights are drawn from a Gaussian distribution (σ = 10% of μ) to produce EPSPs of on average 0.11 mV peak amplitude in exc. and 0.28 mV peak amplitude in inh. neurons at *V*_rest_ Inh. synaptic weights are determined by the factor *g*:

$$g=\frac{J^{i}\tau^{i}\left|V_{\text{rest}}-V_{\text{rev}}^{i}\right|}{J^{e}\tau^{e}\left|V_{\text{rest}}-V_{\text{rev}}^{e}\right|}.$$

We explore the dynamical phase space via numerical network simulations using NEST and PyNN (Gewaltig and Diesmann, 2007). In order to adjust all free parameters we performed a series of exploratory simulation runs. Finally, simulations were performed^3^ for experiments of duration 2 s with varying input parameters g and ν: for all networks, g ranged from 2.5 to 6 (in steps of 0.5) while the input rate variations changed with respect to the connectivity assumptions, see Table 1. Similarly, depending on the network structure, additional simulations were performed for zoom-in-values of g, also listed in Table 1.

**Table 1:** **List of input parameters used to simulate the phase spaces of the five network architectures**.

**Network structures**	**LO**	**OP, PB, RM**	**RD**
Range of ν [KHz]	9.3, 9.4…9.9	9.5, 9.6…10.1	9.25, 9.5…12.0
Additional *g* values	3.17, 3.33	2.67, 2.83	

ν gives the average number of spikes each neuron receives per second from a Poissonian input generator process, and g describes the ratio between exc. and inh. synaptic weights.

The values were adjusted in such a way that each phase space represents all observed dynamical states for each specific network model, as well as the transitions from one to another. The upper and lower boundaries of the input parameter variation were reached if there were no more changes for larger or smaller values of g and ν. Similar to Kumar et al. (2008a,b), Brunel (2000), we neglect inh. inputs and assume them integrated into the external exc. rate.

Since both the connectivity and the simulation of the network dynamics are based on random processes (e.g., random distributions, Poissonian input), we performed a second series of simulations for different network realizations (i.e., with different spatial neuron positions). The results of this control are only mentioned in case of any major deviations between the first and the second simulation series.

### 3.1. Analyzing network dynamics

In order to describe the neurons' activities and their dynamical state, we compute the following observables: the mean firing rate per neuron *FR* (based on time bins of 1 ms length), the mean *free* membrane potential *V*^*e*^_*m*_ (Kuhn et al., 2004), and the mean change in total conductance *G*^*e*^_tot/rest_. In addition, we calculate the typical measures used to characterize a network's phase space: the correlation coefficient *CC* to classify synchronous versus asynchronous spiking, and a specific version of the coefficient of variation *CV* in order to characterize the (ir)regularity in spiking (Brunel, 2000; Kumar et al., 2008a; Voges and Perrinet, 2010). For *FR, CC*, and *CV* we average across exc. and inh. populations (Voges and Perrinet, 2010). We estimate *CC* for time bins of 2 ms, averaging over *N* = 49, 163 (disjoint, randomly chosen) pairs:

(6) $$CC(n_{i},n_{j})=\mathrm{cov}(n_{i},n_{j})/\sqrt{\mathrm{var}(n_{i})\mathrm{var}(n_{j})},$$

where *n*_*i*_, *n*_*j*_ are the spike counts of neuron *i* and *j*, cov denotes their covariance, and var the variance. The *CV* measure used in this article is based on the Kullback–Leibler divergence, and was introduced in (Koyama and Shinomoto, 2007; Voges and Perrinet, 2010):

(7) $$\begin{array}{ccc} CV_{KL}:=\exp(-KL) & \text{with} & KL=\sum \text{P}(\text{ISI})\ln\left[\text{P}(\text{ISI})/\text{Q}(\text{ISI})\right] \end{array}$$

where P(ISI) is the unknown (measured) Inter-Spike-Interval distribution of all neurons, using as reference distribution the exponential Q(ISI) produced by Poissonian spike trains. The advantages of this regularity measure are firstly that it also works for bimodal ISI distribution, and secondly, that it also works in case of very sparse spiking since it is directly determined from the population activity of all neurons (Voges et al., 2010a). To avoid transient effects, the first 500 ms of each simulation run are excluded from the analysis.

Since many of the dynamical states observed for structured networks (**Section 4.1**) are not (completely) congruent to those described for RD networks, we employ two modified measures. First, motivated by our distance-dependent connectivity assumptions, we compute a distance-dependent version of the correlation coefficient, *CC*(*d*), see also Kriener et al. (2009); Yger et al. (2011). To this end, the spatial distances between the pairs of neurons for which *CC* is calculated are sorted into bins of 0.1 mm. Second, in order to capture the spatio-temporal propagation of neuronal spiking, we calculate another extended version of the correlation coefficient, *CC*(*d*_*x*_/τ) and *CC*(*d*_*y*_/τ). For each pair of neurons located at a distance $d=\sqrt{d_{x}+d_{y}}$, *CC*(τ) is determined for varying time delays τ, in steps of 2 ms. Then, *CC*(*d*_*x*,*y*_/τ) is plotted in dependence of the velocities *d*_*x*,*y*_/τ.

**Figure 2:**
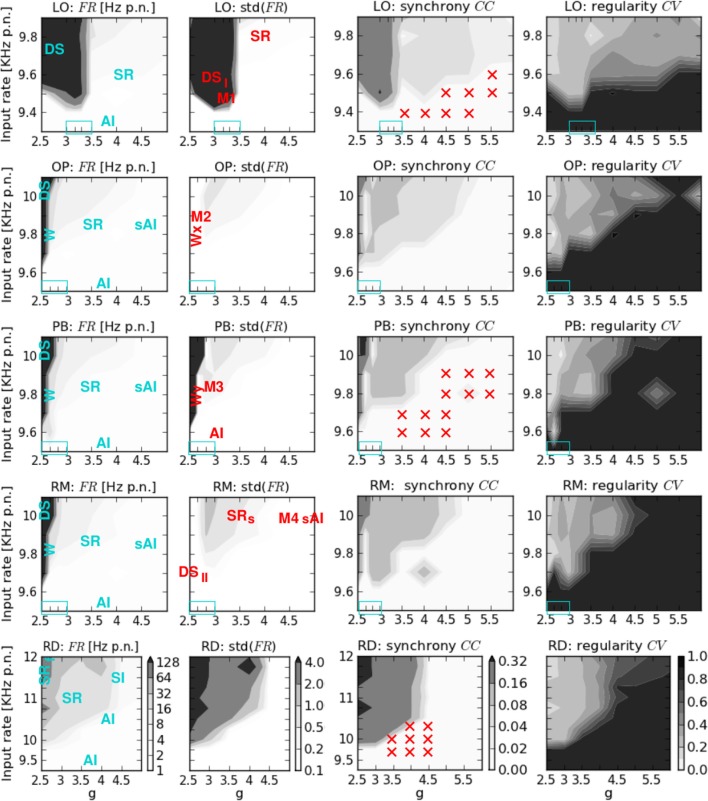
**Phase space analysis of the spiking activity of the five network structures.** From left to right: av. firing rate per neuron *FR* and its standard deviation, av. correlation coefficient *CC*, and coefficient of variation *CV*. From top to bottom: locally connected network (LO), two networks with combined local and patchy remote connections (OP, PB), a network with local aand random remote connections (RM), and a randomly connected network (RD). On the x-axis *g* denotes the ratio between exc. and inh. synaptic weights, while ν on the y-axis indicates the input rate. The colorbars shown at the bottom (RD) are valid for all plots in the corresponding column. Blue text in the *FR* plots describes the states that occur at the corresponding phase space positions. The transition regions (zoom-in values for *g*) are indicated by the blue boxes in the lower left. Red text in the std(FR) plots describes the positions of the specific dynamical patterns shown in Figures 3, 4, and 5. Red crosses in the *CC* plots indicate the input parameter combinations for which additional activity injections were applied (see Table 2).

In addition to the phase space analysis described above, we also examine dynamics subject to spatially and temporally restricted activity injections. Given the network dynamics were classified as AI-like (i.e., asynchronous-irregular), we selected some phase space positions where groups of exc. neurons located near the center of our 2d sheet of cortex receive additional input. The injections start at *t* = 750 ms and stop at *t* = 1500 ms. The synaptic weight distribution is identical to the one assumed for intrinsic connections, while the synaptic delays are 0.2 + 0.02 ms, i.e., small compared to the intrinsic delays. Depending on the specific form of the unperturbed phase spaces, these injections are applied for different *g* and ν values, listed in Table 2, adjusted to hit the corresponding AI states, see Figure 2. We inject with two different additional Poissonian input rates ν_*i*_ = ν × 0.3 and ν_*i*_ = ν × 0.6, respectively, i.e., 30% or 60% of the input rate at the corresponding phase space position. We examine two different injection sizes with a diameter of Ø = 0.3 mm and Ø = 0.6 mm, respectively, i.e., approximately 110 or 430 exc. neurons receive additional input.

**Table 2:** **Phase space positions and resulting dynamics of additional localized activity injections into LO, PB, and RD networks**.

**LO**										
	***g* = 3.5**		***g* = 4.0**		***g* = 4.5**		***g* = 5.0**		**5.5**	
	**ν_*i*_ = 0.3**	**ν_*i*_ = 0.6**	**ν_*i*_ = 0.3**	**ν_*i*_ = 0.6**	**ν_*i*_ = 0.3**	**ν_*i*_ = 0.6**	**ν_*i*_ = 0.3**	**ν_*i*_ = 0.6**	**ν_*i*_ = 0.3**	**ν_*i*_ = 0.6**
ν = 9.6, Ø = 0.6	–	–	–	–	–	–	–	–	SR,M^*p*^	SR
ν = 9.6, Ø = 0.3	–	–	–	–	–	–	–	–	SR	SR
ν = 9.5, Ø = 0.6	–	–	–	–	SR	M^*p*^	SR	SR	M,Wy^*p*^	SR
ν = 9.5, Ø = 0.3	–	–	–	–	SR	SR	SR	SR	SR	SR
ν = 9.4, Ø = 0.6	M^*p*^	M^*p*^	SR	SR	SR	M^*p*^	SR	SR	–	–
ν = 9.4, Ø = 0.3	SR	SR/M^*p*^	SR	SR	SR	SR	SR	M,Wx^*p*^	–	–
**PB**										
ν = 9.9, Ø = 0.6	–	–	–	–	SR	SR	SR	SR/M	SR/M	SR/SI/M
ν = 9.9, Ø = 0.3	–	–	–	–	SR	SR/sAI	SR/SI/M	SR/SI/M	SR_1_/SI	sAI
ν = 9.8, Ø = 0.6	–	–	–	–	SR	SR	SR	SR	SR/M	SR/M
ν = 9.8, Ø = 0.3	–	–	–	–	SR/SI	SR/sAI	SR	sAI	sAI	AI
ν = 9.7, Ø = 0.6	SR	SR	SR	SR	SR	SR/SI	–	–	–	–
ν = 9.7, Ø = 0.3	SR	SR	SR/SI	SR_1_/sAI	SR/SI	SR_1_/sAI	–	–	–	–
ν = 9.6, Ø = 0.6	SR	SR	SR	SR	SR	SR/SI	–	–	–	–
ν = 9.6, Ø = 0.3	SR	SR/SI	SR	SR_1_/sAI	SR	SR_1_/sAI	–	–	–	–
**RD**										
ν = 10.25, Ø = 0.6	–	–	SR_*s*_	SR	SI	SR	–	–	–	–
ν = 10.25, Ø = 0.3	–	–	AI/SI	SI	AI/SI	SI	–	–	–	–
ν = 10, Ø = 0.6	SR	SR	SI	SR	SI	SR	–	–	–	–
ν = 10, Ø = 0.3	SI	SI	AI/SI	AI/SI	AI/SI	SI	–	–	–	–
ν = 9.75, Ø = 0.6	AI	SR	AI/SI	SR	AI	SI	–	–	–	–
ν = 9.75, Ø = 0.3	AI	AI/SI	AI	SR_*s*_	AI	AI	–	–	–	–

All injections are spatially restricted to a radius of either Ø = 0.6 or 0.3 mm with a Poissonian spike rate of either *v*_*i*_ = 0.3 v or 0.6 v. Injections are always applied in the (s)AI state, see Figure 2. The entries indicate the induced activity pattern. The index p indicates a permanent change. Apart from the well-known SR, SI, and AI states, we also observed W (plane waves), M (mixed dynamical patterns, e.g., overlapping waves), a stripy AI state (sAI), and several mixtures between different states (e.g., SR/SI, AI/SI), cf. Figure 7. SR_1_/sAI indicates one burst in an otherwise sAI state.

## 4. Results

We first present the phase spaces resulting from our simulations. Then, we proceed to a more detailed inspection of single dynamical states and their activity patterns (**Section 4.1**). From there, we turn back to the global comparison of the dynamics of different network structures, including the results of localized activity injections (**Section 4.2**).

**Figure A1:**
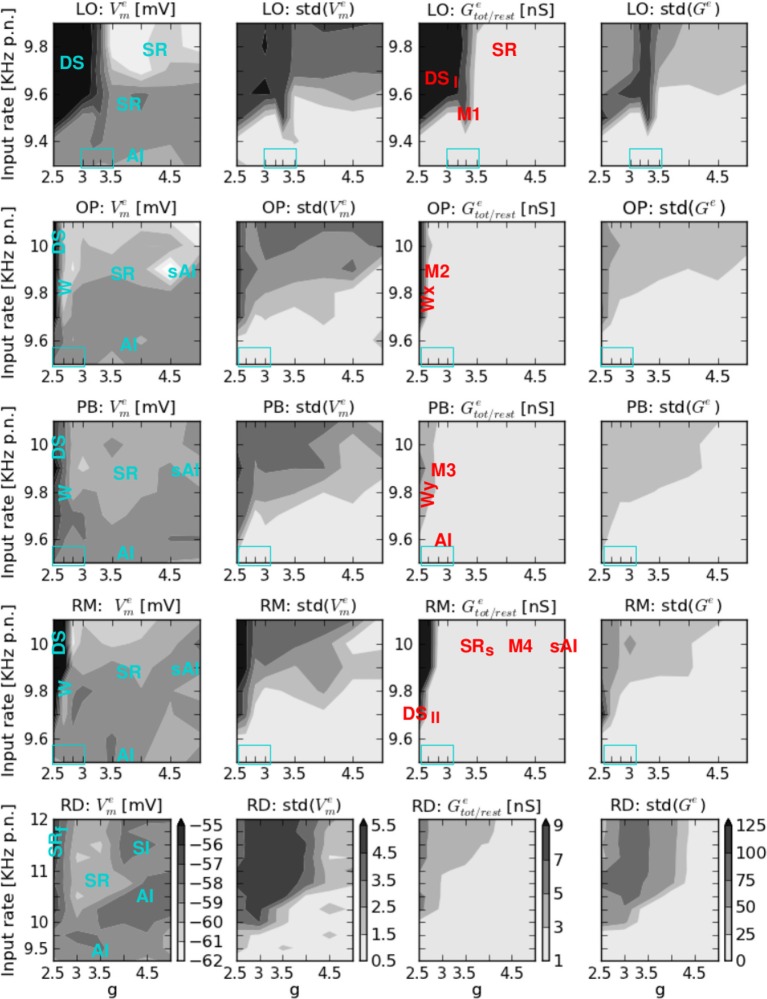
**Phase space, part II.** From left to right: mean free membrane potential *V*^*e*^_*m*_ of the exc. population and its standard deviation, mean change from resting to total conductance *G*^*e*^ of the exc. population, and the variations in the measured *G*^*e*^ values. From top to bottom: LO, OP, PB, RM, and RD network. The colorbars shown at the bottom (RD) are each valid for all plots in the corresponding column. On the x-axis *g* denotes the ratio between exc. and inh. synaptic weights, while ν on the y-axis indicates the input rate. Blue text in the *V*^*e*^_*m*_ plots describes the general states that occur at the corresponding phase space positions. The transition regions (zoom-in values for *g*) are indicated by the blue boxes in the lower left. Red text in the *G*^*e*^_tot/rest_ plots describes the positions of the specific dynamical patterns shown in Figures 3, 4, and 5.

Figure 2 shows the average firing rate *FR* and its standard deviation, the average correlation coefficient *CC*, and the coefficient of variation *CV* obtained by varying the input parameters *g* and ν for the five network architectures considered in this study. We here focus on the measures of the spiking activity, while the results of computing the mean free membrane potential and the mean changes in conductance are presented in Figure A1, see Appendix. The maximum values of these observables and measures are given in Table 3.

**Table 3:** **Maximum values of the phase space observables (mean firing rate *FR*, mean free membrane potential *V*^*e*^_*m*_, mean change from resting to total conductance *G*^*e*^_tot/rest_, and the corresponding standard deviations) and measures (*CC* and *CV*) presented in Figures 2 and A1**.

**Network structures**	**LO**	**OP**	**PB**	**RM**	**RD**
max{*FR*} [Hz p. n.]	253.5	265.1	254.0	261.0	97.2
max{std(*FR*)}	17.55	0.7	27.78	1.17	43.78
max{*CC*}	0.35	0.22	0.35	0.22	0.63
min, max{*V*^*e*^_*m*_} [mV]	−62.4, −51.6	−63.2, −52.4	−60.2, −53.5	−61.3, −52.2	−60.2, −56.05
max{std(*V*^*e*^_*m*_)}	5.61	5.7	5.32	5.9	5.19
max{*G*^*e*^_tot/rest_} [nS]	12.33	12.67	8.18	12.78	6.27
max{std(*G*^*e*^)}	128.6	88.5	63.6	151.0	88.0

For FR, CC, and CV we averaged over exc. and inh. populations while *V*^*e*^_*m*_ and *G*^*e*^_tot/rest_ are given with respect to the exc. population only.

The general structure of the five phase spaces is relatively similar. All networks comprise different regimes characterized by their average firing rates and their amount of synchronicity and regularity in neuronal spiking. The highest *FR* and *CC* values occur for the lowest excitation-inhibition ratios (*g* < *g*_*c*_) and large input rates (ν » ν_*c*_). Likewise, the *CV* is always lowest for *g* < *g*_*c*_ if ν > ν_*c*_. Given a minimum input rate ν > ν_*c*_, the transitions between different regimes depend on both ν and *g*. For input rates below ν_*c*_ we always observe weak irregular asynchronous (AI) firing.

However, the details in the transitions clearly depend on the network structure. They occur with varying smoothness at different phase space positions. For example, in networks including distance-dependent connections (structured networks), the transition from extremely high to lower firing rates is much sharper than for RD networks (cf. Table 3). Therefore, we zoomed in on additional values for *g*, in dependence of the network structure (light blue rectangles in Figure 2, listed in Table 1). With respect to the latter we assumed different ranges for ν which are also listed in Table 1. With respect to these *g* and ν values and the corresponding transition regions, we assign the five phase spaces to three different categories:

- RD networks with a first transition at *g*_*c*_ = 2.5 for $\nu_{c}\underset{˜}{>}10$ KHz separating highly synchronized, regular and strong spiking from medium *FR*, *CC*, and *CV* values. A second transition to asynchronous and irregular spiking occurs for $3<g\underset{˜}{>}4.5$, although firing remains slightly synchronous for $\nu \underset{˜}{>}10.75$ KHz (Voges and Perrinet, 2010).
- Networks with combined local and remote couplings (mixed connectivities OP, PB, RM): a first transition regime appears at *g*_*c*_ ≈ 2.67 for $\nu_{c}\underset{˜}{>}9.7$ KHz, shifted to *g* ≈ 2.83 for $\nu \underset{˜}{>}9.9$ KHz. It separates extremely high (*FR* > 200 KHz) from medium firing rates (7–25 KHz) along with a decrease in synchrony and regularity. In particular, it contains a small range with very low *CC* values. A second transition takes place for $3.5\underset{˜}{<}g\underset{˜}{<}4.5$, depending on the specific ν value (which is higher for larger *g*).
- LO networks with a transition for *g*_*c*_ ≈ 3.3 and $\nu_{c}\underset{˜}{<}9.5$ KHz separating two synchronous regular regimes, one with extremely high firing rates (*FR* > 200 KHz), the other with low to moderate *FR* values (2–20 KHz).

As expected, the network architecture has an effect on the phase space structure, although this effect mainly depends on the ratio between local distance-dependent versus remote connections: the more local Gaussian couplings, the higher the *g* values (i.e., stronger inh. synaptic weights) at which the transitions from strong, synchronous, and regular firing to weak, asynchronous, and irregular spiking take place. Likewise, the more local couplings, the smaller the critical input rate ν_*c*_ which separates low AI firing from the possibility of strong, synchronous-regular spiking. Yet, there is no significant difference in the phase spaces of the mixed connectivity structures (OP, PB, RM), apart from one apparent exception, see Appendix. For a given percentage of local versus long-range connections, the transitions seem independent of the details in the remote connectivity assumptions. These findings are summarized in Figure 8.

In order to compare and characterize the dynamical states themselves, we now present characteristic examples of the activity patterns that occur at different phase space positions.

### 4.1. Complex activity patterns

**Figure 3:**
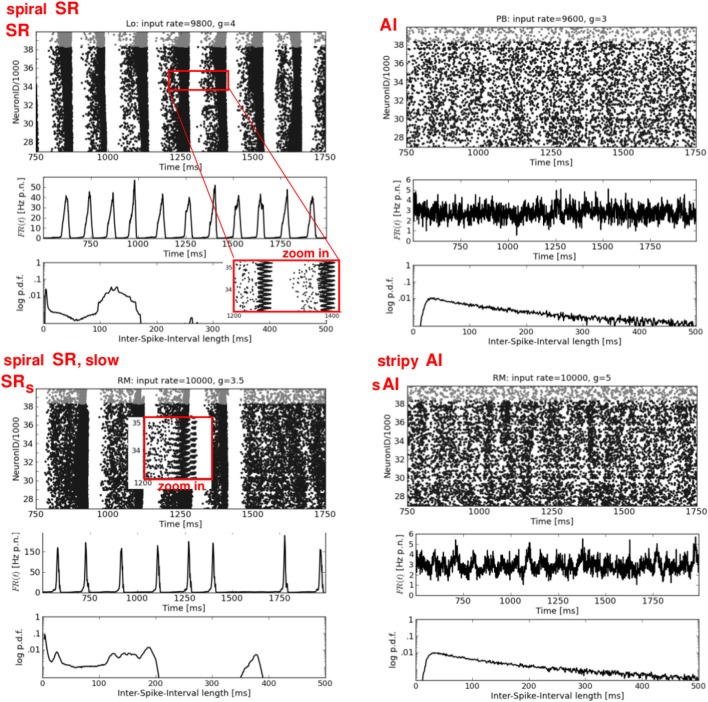
**Synchronous-regular and asynchronous-irregular firing.** Four exemplary raster plots (each black dot represents a spike of one neuron at one time step, top rows) with the corresponding firing rates over time *FR*(*t*) (av. firing rate per neuron in Hz, middle rows) and ISI distributions (logarithmic y-axis, bottom rows). **Left:** two characteristic types of SR activity. **Right:** a typical AI state (top) and a modification of the typical AI with short periods of increased firing of subgroups of neurons, called stripy AI (bottom). The network structure and the input parameters (ν, *g*) used to generate these activity patterns are stated the top of each raster plot.

**Figure 4:**
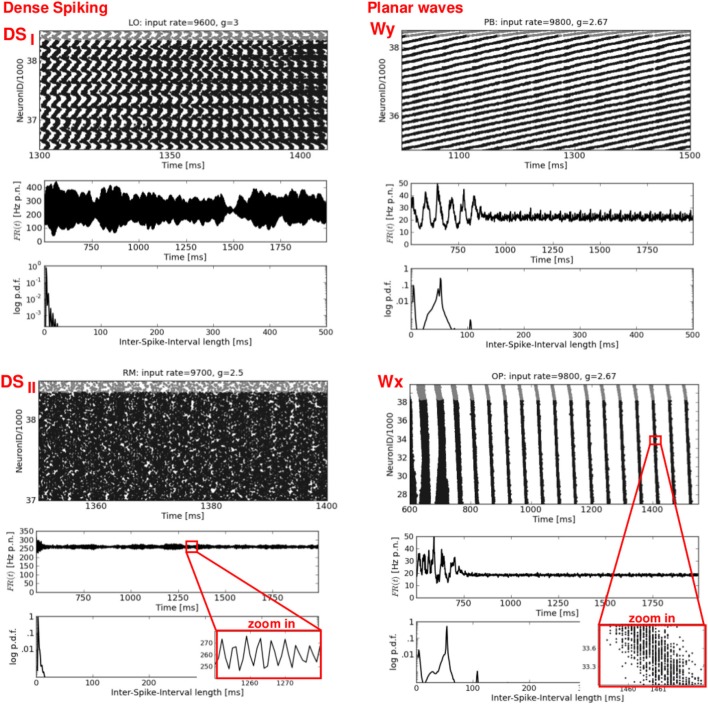
**Dense spiking and plane waves.** Four exemplary raster plots (top rows, please note the different time scales) with the corresponding *FR*(*t*) curves (middle rows) and ISI distributions (bottom rows). **Left:** two types of dense spiking DS, characterized by exceptionally high firing rates. **Right:** plane waves beginning after approximately 750–900 ms. Note the flat *FR*(*t*). Thin oblique horizontal stripes indicate a propagation in y-direction (see Figure 6, top row), while thin oblique vertical stripes indicate a propagation in x-direction. Again, the network structures and the input parameters used to generate these activity patterns are stated at the top of each raster plot.

**Figure 5:**
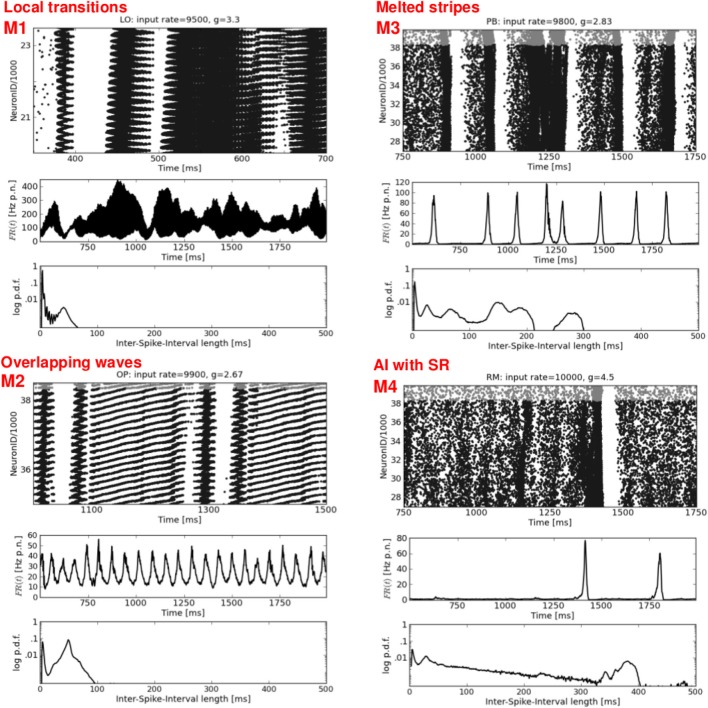
**Mixed spike patterns.** Four exemplary raster plots (top rows, please note the different time scales) with the corresponding *FR*(*t*) curves (middle rows) and ISI distributions (bottom rows). **M1** occurs in LO networks and indicates a mixture between DS,SR, and plane waves. **M2** represents an ongoing change between SR and Wy. **M3** shows a typical variation of SR activity in structured networks: melting bursts.(at *t* ≈ 1250 ms), and short periods of increased firing shortly before an actual burst occurs (at *t* ≈ 1400 and 1600 ms). **M4** shows a typical activity pattern, namely one single SR burst embedded in an otherwise sAI state. Network structure and input parameters used to generate these activity patterns are stated at the top of each raster plot.

Figures 3, 4, and 5 show the raster plots, the firing rate over time, and the ISI distributions for the phase space positions marked in Figure 2. These activity patterns are representative for the various states resulting from simulating the dynamics of structured networks, i.e., LO, OP, PB and RM network models.

**Figure 6:**
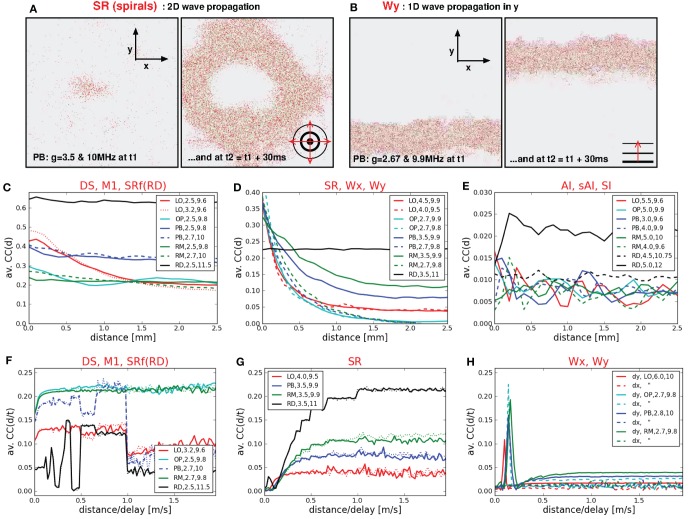
**Spatio-temporal activity propagation. (A,B)** Two 2d sheets of cortex, each at two different time steps. Each dot represents the position of a spike of a single neuron. **(A)** Shows a circular spherical wave propagation while **(B)** shows a plane wave of spikes that propagates only in y-direction. **(C,D,E,F,G,H)** Distance-dependent and velocity-dependent average correlation coefficients, subdivided into different categories according to the dynamical state. **(C,F)** *CC*(*d*) and *CC*(*d*/τ) for states with extremely high firing rates (DS, M3, SR of RD networks), **(D)** *CC*(*d*) for states with medium to high firing rates (SR, Wx, Wy), **(E)** *CC*(*d*) for states with low firing rates, **(G)** *CC*(*d*/τ) for 2d waves, **(H)** *CC*(*d*/τ) for 1d waves.

Figure 3 shows two examples each of synchronous-regular (SR) and asynchronous-irregular (AI) firing. Similar to what we found for RD networks (Voges and Perrinet, 2010), the SR states are not as clearly defined as it has previously been reported (Brunel, 2000; Kumar et al., 2008a). A burst of spikes starts with a shorter (Figure 3, top left) or longer (Figure 3, bottom left) interval of AI firing which initiates a short period of synchronous-regular spiking involving (almost) all neurons [*FR*(*t*) maxima], followed by a refractory period without any spikes. We distinguish between SR and SR_*s*_ (i.e., slow) with respect to the frequency of bursts. The higher the input rate ν and the smaller *g* (lower inh. weights) the more bursts occur—with less AI firing in between. The corresponding ISI distributions are either bimodal (periodic bursting, high *CV*) or even multi-modal in case of irregularly appearing bumps (intermediate *CV*). A zoom-in on the raster plots, however, reveals a clear difference between the SR states of structured versus random networks: for networks with local distance-dependent connections, each burst exhibits a spiral structure, indicating a spherical wave in the 2d geometry of our sheet of cortex^4^, see Figure 6, top left. A small spot of activity emerges at some random position, it spreads out circularly over the whole spatial domain, and then vanishes (Schmidt et al., 2010; Yger et al., 2011). In the RD network there is no such spatial propagation. The corresponding 2d view of SR activity in the RD model is a synchronous blinking of (almost) all neurons.

The right column of Figure 3 shows two types of weak asynchronous-irregular activity that occur in structured networks. The upper plot is a typical AI state, well-known from RD networks (Brunel, 2000; Kumar et al., 2008a; Voges and Perrinet, 2010): a low and flat *FR*(*t*) together with an exponential ISI distribution. In our structured networks, a pure AI state appears only rarely, mainly for ν < ν_*c*_. Mostly, we find so-called stripy AI (sAI) dynamics, see Figure 3, bottom right. We see principally asynchronous-irregular firing [with a still relatively flat *FR*(*t*)] that contains small stripes of clustered spikes (Kriener et al., 2009). Yet, these spatio-temporal clusters of spikes are not pronounced enough to establish a proper SI state: they involve only subgroups of all neurons so that $CC\underset{˜}{<}0.002$ at the corresponding phase space locations (Figure 2). This is even lower than the correlations defining the weak SI state in RD networks, namely $0.003\underset{˜}{<}CC\underset{˜}{<}0.01$, see Voges and Perrinet (2010).

The left column of Figure 4 shows two types of dense spiking with exceptionally high firing rates and a pronounced peak at very short ISIs that occur in structured networks for ν > ν_*c*_ and *g* < *g*_*c*_. The corresponding activity in RD networks consists of thin vertical stripes due to highly synchronous firing of all neurons, called SR_fast_ (Kumar et al., 2008a; Voges and Perrinet, 2010). Instead, structured networks exhibit either chessboard-like raster plots together with exceptionally strong *FR*(*t*) fluctuations called DS_*I*_, or a state named DS_*II*_ with smaller, very fast *FR*(*t*) fluctuations. This difference between DS_*I*_ and DS_*II*_ may be seen in the phase space plots where we have low std(*FR*) values at ν > ν_*c*_ and *g* < *g*_*c*_ for OP and RM networks compared to LO and PB networks (Figure 2).

The right column of Figure 4 presents a second type of waves that occurs mainly for mixed network structures (OP, PB, RM) at *g* ≈ 2.67 or *g* ≈ 2.83, i.e., at the border between DS and SR activity. A raster plot with thin, oblique, horizontally oriented stripes (top right) indicates a plane wave that propagates in y-direction, while thin, oblique, vertically oriented stripes (bottom right) indicate a plane wave that propagates in x-direction. Figure 6, top right gives an example of such a wave propagating in y, from the bottom to the top of our 2d sheet (then re-entering at the bottom due to the periodic boundary conditions). Plane waves exhibit a bimodal ISI distribution (small *CV*, i.e., regular spiking) but a flat *FR*(*t*), which is the reason for the small *CC* values in Figure 2. They emerge after a certain settling time, often due to interference of several spherical waves, cf. M3 in Figure 5. These states are rather transient, mostly mixed with SR or DS activity patterns, see M2 in Figure 5. They often appear as part of an ongoing state change (called “local transition”) in LO networks, see Figure 5, top left. Other transient or mixed states are, for example, M2 in Figure 5, bottom left which indicates a periodic switching between plane and spherical waves. Melting stripes (M3 in Figure 5, top right) sometimes induce a transition into another activity pattern. M4 shows a typical mixture between sAI, SR, and SI activity which usually appears at the border between SR and sAI states.

In general, the typical measures used to characterize spiking activity of random networks *FR*, *CC*, and *CV* are still appropriate for structured networks. There are, however, new or modified activity patterns that do not exist in RD networks, as, for example, propagating waves or dense spiking. Some of them are not adequately described by the usual measures (see also **Section 4.2**), e.g., the average *CC* in case of plane waves: intuitively, spherical and plane waves are just two realizations of one and the same activity pattern. Yet, the flat *FR*(*t*) in Figure 4, as well as the low *CC* values in Figure 2 indicate asynchronous firing for Wx and Wy states, whereas spherical waves are clearly classified as SR. Thus, in particular to capture the spatio-temporal propagation of plane waves, we compute *CC*(*d*) and *CC*(τ/*d*) (middle and bottom row of Figure 6).

We find that the correlation coefficients of states with very high firing rates (DS, M1 in LO networks, SR_*f*_ in RD networks) depend only weakly on the spatial distances (Figure 6C). Only in case of purely local couplings *CC*(*d*) clearly decreases (red curve). The *CC*(*d*) values of the RD network were highest and independent of *d*. Figure 6D shows *CC*(*d*) for states with high or intermediate firing rates. These curves decrease with increasing *d* for structured networks, only the RD model remains independent of *d* (black line). Thus, in case of intermediate *FR* values, a higher connection probability between neighboring neurons leads to a more synchronized firing of those neurons (cf. Kriener et al., 2009; Yger et al., 2011). Yet, there is no difference between SR states with one single burst (dotted red line) or several bursts (e.g., continuous red line). Moreover, this measure does not distinguish between plane and spherical waves, although their average *CC* values differ significantly. As mentioned above, plane waves (cyan lines and dotted green line) are usually intermingled with SR or DS activity patterns and one single burst is enough to induce a *CC*(*d*) curve similar to frequently bursting SR dynamics. Likewise, *CC*(*d*) yields similar curves for all states with low firing rates (Figure 6E), independent of the network structure (cf. Yger et al., 2011). Only the SI state of RD networks exhibits slightly higher *CC*(*d*) values due to a slightly higher average *CC* value (Voges and Perrinet, 2010). Finally, by calculating *CC*(*d*_*x*_/τ) and *CC*(*d*_*y*_/τ), we are able to identify the spatio-temporal propagation of plane waves. The peaks in Figure 6H clearly indicate such waves traveling in x- or y-direction. More precisely, they indicate velocities of approximately 0.1–0.2 m/s.

### 4.2. Phase space analysis and activity injections

We have demonstrated that local distance-dependent connections lead to new, more complex activity patterns that do not occur for purely random couplings. In particular, structured networks with distance-dependent conduction delays induce a spatial spread of spiking activity, i.e., plane or spherical waves (Schmidt et al., 2010; Yger et al., 2011). Comparing the phase space of our networks we find that the correlation coefficient is generally higher in the RD model (Figure 2). On one hand, this might be an effect of the spatio-temporal activity propagation since Equation (6) does not allow for any spatial properties, and we did not adapt the bin size^5^. On the other hand, Renart et al. (2010) demonstrate that substantial amounts of shared input (as caused by neighborhood couplings) do not necessarily lead to increased correlations. Moreover, Ecker et al. (2010) suggests an active decorrelation of adjacent neurons. We also find that the distribution of *FR* values was much smoother for RD networks (Figure 2), whereas the maximum firing rates are extremely high for structured networks, see Table 3. Most likely, this is due to the increased recurrency in networks with distance-dependent couplings leading to a mutual activation of neighboring neurons (positive feedback loop). However, it seems that this feature is not graduated in terms of the amount of local couplings since the maximum *FR* values of LO, OP, PB, and LO are quite similar.

Our analysis reveals that some states occur only for certain network structures, or at specific phase space locations depending on the network type. Plane waves, for example, appear either at the border between DS and SR activity in networks with mixed connectivities (*g* = 2.67, 2.83 for OP, PB, RM), or for *g* ≈ 6 in LO networks. None of our network models shows a clear SI state. Instead, we obtain a modified AI state containing fragmentary stripes of spikes (sAI). Additionally, there are many transient regimes, i.e., mixed states in which the activity pattern changes between different types of waves, or in which one or two occasional bursts appear during an otherwise (s)AI firing. Such a coexistence of two (or more) states occurs mostly at the borders between DS and SR spiking (Figure 5) or between SR and (s)AI firing, similar to what we found for RD networks (Voges and Perrinet, 2010). Therefore, with respect to the question of stability, we perform a series of simulations that involve additional, localized activity injections, listed in Table 2. Since the results for networks with mixed connectivities (OP, PB and RM model) are qualitatively identical, we only present PB injections.

**Figure 7:**
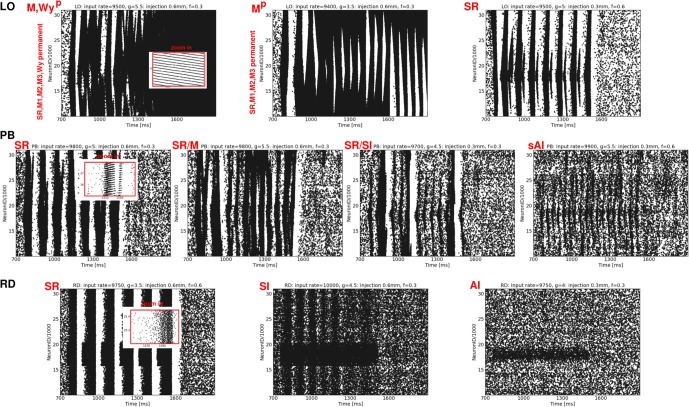
**Exemplary raster plots resulting from additional localized activity injections** (each black dot represents a spike of one neuron at one time step). **Top row:** LO network, **middle row:** PB network, **bottom row**: RD network. The labeling of the resulting states listed in Table 2 is given in red. The ν, *g*, ν_*i*_, and Ø values used to generate these activity patterns are given at the top of each plot.

In order to enable the possibility of state change we restrict the injections to exc. neurons. Targeting inh. neurons always results in a clear reduction of neuronal firing, independent of the network type. Yet, this is not in agreement with cortical reality for input coming from the thalamus (Kremkow et al., 2010) and, therefore, indicates an issue with the general set-up of our networks (as discussed later on). Figure 7 shows a selection of raster plots of the activity patterns resulting from localized injections, ordered according to the network structure. These plots are chosen to be representative examples of the states listed in Table 2.

Depending on both the injection parameters and the network architecture, the dynamical state may switch from (s)AI firing to SR or W activity or to states with extremely high firing rates (SD, M). Mostly, the activity switches back to the original (s)AI state as soon as the injection ends. Only in case of LO networks do some injections induce a permanent state change (indicated by an upper index *p*), mainly, but not exclusively, for injections at phase space positions with small *g* values (Table 2). Likewise, only in case of LO networks, do all injections lead to a total change of the activity dynamics, i.e., to traveling waves (SR, W) or mixtures of states with extremely high firing rates. In contrast, for RD models or networks with mixed connectivities, neuronal firing can also remain weak and irregular, see Figure 7, left and Table 2, bottom—provided that ν is not too large and *g* is large enough. Additional activity in the RD network at small ν may even remain locally confined to the injection site (Figure 7, bottom right). In the PB network, additional spikes always propagate to neighboring neurons, but usually not across the whole network, i.e., injection usually does not induce SI activity. If so, this occurs typically in combination with one or two SR bursts (Table 2). Principally, the phase space position of the injection has the strongest effect: the smaller *g* and the larger ν the higher the probability for a major change, i.e., a transition to some type of regular state (SR firing, plane waves or a mixture between these states). The injection parameters are less important, the impact of Ø is typically larger than the impact of ν_*i*_. Quantitatively, both parameters operate in the same direction as ν. In summary, we see that sensitivity to additional input clearly increases from RD networks to networks with mixed connectivities to LO networks, which exhibit particularly unstable, at times even permanently changed, dynamics.

## 5. Discussion and conclusion

Aiming to shed light on the effect of different intrinsic connectivity assumption on cortical network dynamics, we analyze the phase spaces of three network categories: (1) purely random couplings as often used in studying cortical network dynamics, (2) purely local couplings as an opposite to random networks, and (3) three types of combined local and remote connectivities. The latter are carefully chosen in order to represent neuroanatomical findings. They differ in terms of the spatial arrangement of their remote synapses: random versus two types of patchy projections.

**Figure 8:**
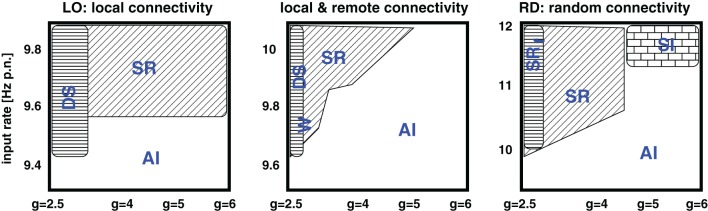
**Schematic summary of Figure 2.** The five phase spaces are assigned into three different categories: Purely local couplings, mixed (i.e., separated local and remote) connectivity assumptions, and purely random couplings. SR indicates synchronous-regular like firing, AI asynchronous-irregular like firing, DS dense spiking, and W the occurence of waves.

We find that different connectivity assumptions lead to shifted phase space transitions, summarized in Figure 8. The critical parameter is the percentage of local distance-dependent couplings versus remotely established synapses (Stepanyants et al., 2009). Details in the spatial arrangement of the remote connections have neither a visible effect on the “idle” dynamics, nor on the activity patterns generated by additional localized Poissonian inputs.

The ratio between local and remote couplings affected not only the transitions between different regimes in the phase space, but also the regimes themselves. Depending on the input parameters, local distance-dependent couplings induce extremely high firing rates. In particular, they cause a spatio-temporal spread of activity, i.e., propagating waves (Schmidt et al., 2010; Yger et al., 2011). Moreover, for input rates larger than a certain critical value, networks with mixed connectivity mainly exhibit a stripy AI state instead of the clear asynchronous irregular (AI) or synchronous irregular (SI) firing described previously (Brunel, 2000; Kumar et al., 2008a; Voges and Perrinet, 2010). For ν > ν_*c*_ the dynamics of purely LO is always composed of waves (regardless of *g*) and, for ν < ν_*c*_, we find them to be rather unstable (**Section 4.2**).

Significant phase space modifications have already been demonstrated for random networks: with respect to conductance-based synapses (Brunel, 2000; Kumar et al., 2008a) and as a consequence of a whole-set of changes in Voges and Perrinet (2010) compared to Brunel (2000); Kumar et al. (2008a). As mentioned before, we use the general set-up described in Voges and Perrinet, 2010 in order to focus on the comparison of different network structures. There are some general issues concerning our networks. First, the spatial enlargement (5 × 5 instead of one squared millimeter) of our models is naturally at the expense of an unrealistically small neuron density (Voges and Perrinet, 2010). Second, we do not consider depressing or facilitating synapses, although this is a known property of cortical neurons (Nowak et al., 2003). Most probably, this is the reason why we have to confine our additional activity injections to exc. neurons: actually, fast spiking neurons often slow down for tonic input. Third, our notion of LO networks deviates from the usual meaning of local networks. The crucial feature of our LO model is that there are many neurons *outside* the local connectivity range, i.e., neurons with which the central neuron establishes no synapses at all, see Figure 1. In contrast, the usual definition implies a non-zero connection probability between any pair of neurons (Mehring et al., 2003; Kumar et al., 2008b; Yger et al., 2011). Since we focus on the impact of explicit remote (as opposed to local) projections, our LO model represents one extreme connectivity assumption, while the RD model represent the other extreme.

A well-known network model that spans the whole range between regular and random connectivity is the small-world network (Newman, 2003; Voges et al., 2010a, 2012b). Kitano and Fukai (2007) analyze the effect of explicit long-range connections on the dynamics of 2d spatially embedded networks by varying the rewiring probability *p*_*w*_. *p*_*w*_ = 1 and *p*_*w*_ = 0 roughly correspond to our RD and LO models, respectively, while our mixed connectivities correspond to *p*_*w*_ = 0.4. Kitano and Fukai (2007) demonstrates a monotonically increasing spiking regularity (small *CV*) as a consequence of decreasing *p*_*w*_, i.e., less long-range connectivity. This is in agreement with our results if one compares the *CV* values for RD and LO networks in Figure 2. Their *p*_*w*_ value to maximize synchronicity was found to depend on the synaptic strength, it was either *p*_*w*_ = 1 in agreement with our findings, or *p*_*w*_ = 0.05. Our models cannot contribute to the discussion as in (Roxin, 2011) as nearly all our degree distributions are binomial (Voges et al., 2010a). There is one exception, the PB model which has a broader out-degree distribution. Yet, this corresponds to a case where Roxin (2011) shows the total *CC* to be unaffected.

Recently, Yger et al. (2011) published a study with respect to local connectivity (in the usual sense). Focusing on the SI regime, they vary the spatial spread of a Gaussian connectivity profile σ_*c*_. They find that the macroscopic properties of the spiking activity are basically invariant with respect to σ_*c*_. Similarly, we claim that details in the remote connectivity have little impact on the network dynamics. Moreover, Yger et al. (2011) state that their findings do not hold in the limit of very small σ_*c*_ which corresponds to our LO network. Yet, these two studies principally represent two different approaches: we systematically vary the input rate ν while Yger et al. (2011) vary σ_*c*_ for a fixed ν. We analyze the transitions between different regimes, whereas they focus on a single regime, the SI state. However, we see no SI, but rather a stripy AI regime instead. On one hand, this might be due to the different parameter values for the single neurons, the conduction delays, etc. On the other hand, it may be an effect of the distinct spatial scales (5 × 5 versus one squared millimeter) resulting in distinct global connectivity assumptions: Our connection density is a factor 3 smaller than their minimum (0.15% versus 0.5%) and we separate between local and remote projections. Thus, our fragmentary stripes, which are indeed synchronized spikes of clusters of neighboring neurons (due to the neuron ordering, see **Section 2.1**), could well be SI firing on a more local spatial scale.

In summary, we demonstrate that the connectivity type assumed for discrete cortical network does play a role in the resulting dynamics (Figure 8). Thus, depending on the aim of the study, one should be aware of the characteristics and limits of the chosen network structure. Random networks, for example, cannot account for propagating waves and are therefore less appropriate to investigate a spatio-temporal spread of activity. Purely LO show no (a)synchronous-regular regime for higher input rates and they are extremely sensitive to additional localized inputs (unstable). The assumption of a Gaussian connectivity profile for spatially extended networks (i.e., including remote intrinsic synapses) is presumably not appropriate. Choosing a σ that correctly captures the local connectivity range of each neuron most probably results in a far too low connection probability for the distant projection targets (similar to our LO model). One solution is to distinguish between local and remote connectivity. Another possibility is to assume another distance-dependent connection probability profile, e.g., one with a slower decay or a heavy tail distribution. As mentioned in **Section 2**, there are indeed studies that suggest an exponential decay for the local connectivity (Holmgren et al., 2003).

In general, heterogeneity is an important property of cortical networks (Denker et al., 2004; Tetzlaff et al., 2009). Our results support this statement with respect to different connectivity types. Moreover, as mentioned in **Section 1**, a mixture of local and remote couplings is crucial with regard to the wiring optimization necessary for real cortical networks (Chklovskii, 2004). Patchy connections provide an additional advantage: they ensure synapses between distant groups of neurons using very little cable length (Voges et al., 2010b). We show that the inclusion of this advantageous feature neither changes the “idle” dynamics, nor the network's reaction to additional localized input. Thus, if one aims at closely representing cortical connectivity, the relatively complex PB model would be a good choice. However, if one simply needs a stable AI state, the RD model should suffice, see also below. Patchy connectivity patterns most probably come into play when information is processed, i.e., with respect to functional aspects like orientation selectivity (Buzás et al., 2006), receptive field properties (Angelucci and Bressloff, 2006), or for embedding synfire chains (Kumar et al., 2010).

Our results are an encouraging step toward a reconciliation of the apparent conflict between detailed topological network models (Mehring et al., 2003; Kumar et al., 2008a,b; Voges et al., 2010a; Yger et al., 2011) and the mean-field approach taken in neural mass models (Jansen and Rit, 1995; Deco et al., 2011; Voges et al., 2012a), similar to those presented by Yger et al., 2011. As mentioned in **Section 1** these macroscopic models often consist of several single units with random internal couplings, representing the neuronal connectivity inside the range of a cortical column (Jansen and Rit, 1995). Then, the units are typically connected by specific external links, representing (white matter) connections between different (functional) areas of the brain (e.g., V1, V2, MT, A1). On one hand, such models neglect details of the spatio-temporal dynamics on the small (neuronal) scale. On the other hand, the activity dynamics in such macroscopic models is rather dominated by the corresponding properties of the external connections (e.g., their conduction speed), in particular with regard to the resting state dynamics (Deco et al., 2011). Thus, such neural mass models are an appropriate approximation as long as the spatial scale is large enough and as long as the properties to be analyzed are sufficiently macroscopic. Yet, concerning the investigation of issues that involve topographic projections (e.g., receptive field properties) or phenomena that might be influenced by spatial aspects (e.g., negative BOLD responses in epilepsy, see Voges et al., 2012a), spatially embedded single-cell networks are a better choice.

Finally, we would like to stress the point that there is a connectivity scale in between the cortical column and white matter projections. Including these remote but mostly intrinsic connections inside one area or between neighboring brain regions has an impact on the phase space dynamics.

### Conflict of interest statement

The authors declare that the research was conducted in the absence of any commercial or financial relationships that could be construed as a potential conflict of interest.

## Footnotes

^1^The neurons are primarily sorted by ascending *y*-coordinate, then by ascending *x*. This means that in our 2d sheet of cortex, neuron zero is located at the top left, whereas neuron 49,162 is located at the bottom right.

^2^Where the sheet of cortex is subdivided into 10 × 10 boxes of side length *bl* = 0.5 mm.

^3^One simulation run, including all parameter variations and the calculation of the resulting measures, took around one week of CPU time.

^4^The spiral is a visualization of the neuron ordering, see **Section 2.1**.

^5^Which was chosen to be 2 ms for all networks, independently of the waves' velocities.

## Appendix

We here present the second part of the phase space, i.e., the mean free membrane potential *V*^*e*^_*m*_, the mean change from resting to total conductance *G*^*e*^_tot/rest_ (each for the exc. population only), and their corresponding standard deviations for varying *g* and ν. Averaging over exc. and inh. neurons is not appropriate for these observables due to different types of neurons; we focus on the exc. population (Voges and Perrinet, 2010).

A comparison between Figure 2 and A1 shows that *FR* (cf. Figure 2), *V*^*e*^_*m*_, *G*^*e*^_tot/rest'_, and their standard deviations exhibit a similar behavior with respect to the input parameters. Maximum values occur for low inhibition combined with high input rates, and minimum values for large *g* and small ν. In case of AI or sAI firing (ν > ν_*c*_ or *g* > *g*_*c*_ together with the corresponding ν values) *V*^*e*^_*m*_ weakly fluctuates [small std(*V*^*e*^_*m*_)] a few millivolts below the threshold *V*_θ_. Membrane conductances are increased by a factor 1.2–3 relative to the membrane conductance at rest for exc. neurons. Correspondingly, this leads to a reduction of τ^*e*^_rest_ =10 ms to 3–8 ms. For regimes with medium to relatively high firing rates (W, SR or SR_*s*_), *V*^*e*^_*m*_ shows slightly lower values but larger fluctuations while the changes in *G*^*e*^_tot/rest_ are similar to those described for the (s)AI state but with larger fluctuations. Thus, these states are in line with *in vivo* physiological observations (Destexh et al., 2003).

However, the dynamical states with exceptionally high *FR* values (DS for ν < ν_*c*_ and *g* < *g*_*c*_ in structured networks) are surely very far from physiological observations in healthy animals. The mean *free* membrane potential can exceed the firing threshold, the increase in membrane conductance exceeds a factor of 10, and the corresponding fluctuations are huge (see Table 3). They are even more pathological than the corresponding SR_f_ state in RD networks (Voges and Perrinet, 2010). Such a regime constitutes a comparably large part of the phase space of the LO network, see Figure 2 and A1. In case of mixed connectivities (OP, PB, and RM network) such a state occurs rarely, only for *g* < 2.67 or *g* < 2.83 together with very high input rates. The apparent difference between the phase spaces of PB versus OP and RM networks results from this pathological regime: the vertical stripe of high std(*FR*) and *CC* values occurs only in case of PB connectivity, at $2.5\underset{˜}{<}g\underset{˜}{<}2.67$ for $\nu \underset{˜}{>}9.7$ KHz. However, this difference is not visible in Figure A1 and there is no such vertical stripe in our second series of simulations. The results of this control are qualitatively identical to the ones shown in Figure 2, apart from the effect described above.
